# Polyamine Oxidase-Generated Reactive Oxygen Species in Plant Development and Adaptation: The Polyamine Oxidase—NADPH Oxidase Nexus

**DOI:** 10.3390/antiox11122488

**Published:** 2022-12-17

**Authors:** Péter Benkő, Katalin Gémes, Attila Fehér

**Affiliations:** 1 Department of Plant Biology, University of Szeged, 52. Közép Fasor, H-6726 Szeged, Hungary; 2 Doctoral School of Biology, University of Szeged, 52. Közép Fasor, H-6726 Szeged, Hungary; 3 Institute of Plant Biology, Biological Research Centre, Hungarian Academy of Sciences, 62. Temesvári Krt, H-6726 Szeged, Hungary

**Keywords:** polyamines, polyamine oxidase, NADPH oxidase, polyamine catabolism, stress response, hydrogen peroxide

## Abstract

Metabolism and regulation of cellular polyamine levels are crucial for living cells to maintain their homeostasis and function. Polyamine oxidases (PAOs) terminally catabolize polyamines or catalyse the back-conversion reactions when spermine is converted to spermidine and Spd to putrescine. Hydrogen peroxide (H_2_O_2_) is a by-product of both the catabolic and back-conversion processes. Pharmacological and genetic approaches have started to uncover the roles of PAO-generated H_2_O_2_ in various plant developmental and adaptation processes such as cell differentiation, senescence, programmed cell death, and abiotic and biotic stress responses. Many of these studies have revealed that the superoxide-generating Respiratory Burst Oxidase Homolog (RBOH) NADPH oxidases control the same processes either upstream or downstream of PAO action. Therefore, it is reasonable to suppose that the two enzymes co-ordinately control the cellular homeostasis of reactive oxygen species. The intricate relationship between PAOs and RBOHs is also discussed, posing the hypothesis that these enzymes indirectly control each other’s abundance/function via H_2_O_2_.

## 1. Introduction

Polyamines (PAs) are small, positively-charged organic molecules that are present in all living organisms. PAs show tissue- and organ-specific distribution patterns [1,2]. The most relevant PAs in plant cells are putrescine (Put), spermidine (Spd), and spermine (Spm). In plants, PA biosynthesis produces Put from arginine catalysed by the arginine decarboxylase enzyme (ADC), but in several plants, the ornithine decarboxylase (ODC) can also synthesise Put from ornithine [2,3,4]. Put can be converted to Spd by spermidine synthase (SPDS), which can be further converted to Spm by spermine synthase (SPMS). Thermospermine (t-Spm) is a specially modified polyamine synthesized by the thermospermine synthase (named ACAULIS5 or ACL5 in Arabidopsis) by transferring an aminopropyl residue to the N- terminal amino group of Spd [5]. Some species also have cadaverin (Cad) which is synthesized from lysine through a fully independent pathway by ornithine/lysine decarboxylases (O/LDCs) [6]. PAs can be found in plant cells in different forms, such as free, covalently conjugated, or non-covalently conjugated ones. The covalently conjugated PAs can be further classified as perchloric acid-soluble or insoluble [2].

PAs are involved in cell division, organ development, leaf senescence, fruit development and ripening, and abiotic stress responses, [2,7,8]. The involvement of PAs in stress tolerance has many aspects. They directly interact with and protect macromolecules and organelle membranes acting as compatible solutes. Further, they (in)directly scavenge oxygen and hydroxyl radicals, promote the production of H_2_O_2_ acting as a signal molecule, thereby contributing to the production of antioxidant enzymes and metabolites, contribute to nitric oxide (NO) production, regulate ion channels and metabolic activities, for example, limit ammonia toxicity [1,8]. Their cellular levels depend on the different phases of plant growth and development and the level and form of environmental stress experienced by the plant [1]. Exogenous applications of PAs often result in higher stress tolerance, but higher than optimal levels prove to be toxic [8]. It is clear, therefore, that the polyamine homeostasis of the cells must be tightly regulated to ensure their proper functioning and adaptation. Neither the direct relationship between increased levels of PAs and abiotic stress tolerance nor the mechanism by which PAs regulate plant growth and stress responses is still fully understood [2,9].

Plants, in response to different stress stimuli but also during normal metabolism, produce reactive oxygen species (ROS). These include oxygen radicals such as superoxide anion (O_2_•-), hydroperoxyl radical (HO_2_), alkoxy radical (RO•), and hydroxyl radical (HO•), as well as nonradicals such as hydrogen peroxide (H_2_O_2_) and singlet oxygen (^1^O_2_). At higher concentrations, ROS can damage cell content, which can lead to programmed cell death [10]. Therefore, their levels must be tightly controlled [11,12]. To avoid harmful ROS accumulation, plants have developed various ROS scavengers [11]. Plants deal with oxidative stress primarily by enzymatic and non-enzymatic antioxidants present in all cellular compartments [12,13]. 

Despite their potentially harmful nature, ROS are involved in the regulation of various metabolic, physiological, and developmental processes [12,14,15,16]. To name a few, ROS are needed for the development of root and shoot apical meristems, the emergence of lateral roots, and the polar growth of root hair cells and pollen tubes. ROS can specifically alter gene expression [16,17] and transmit information about changing environmental conditions [12,15,16]. Nevertheless, we hardly know about the perception of ROS and the immediate downstream elements of their signalling [11]. ROS can act locally but can also spread from the place of synthesis [18]. Among ROS, H_2_O_2_ has the longest half-life (ca. 1ms) and thus can signal from the longest distance, even between cells in the apoplast [19].

In plants, the NADPH oxidases (NOX), also called respiratory burst oxidase homologs (RBOHs), are located in the plasma membrane and contribute to apoplastic H_2_O_2_ accumulation. Their enzymatic activity catalyses the production of apoplastic O_2_•- by transferring electrons from cytosolic NADPH or NADH to apoplastic O_2_. O_2_•- is further converted to H_2_O_2_ by superoxide dismutases [20]. NADPH oxidases are involved in abiotic and biotic stress responses and various aspects of plant development [13,20]. In *Arabidopsis thaliana*, ten isoforms of the NADPH oxidase enzyme have been identified. They are named RBOH (A–J), of which RBOHD and RBOHF play crucial roles in both biotic and abiotic stress responses. Others, such as RBOHC, RBOHH, and RBOHJ, are rather related to plant developmental processes [21].

Along with other flavoenzymes, such as the NADPH oxidases and xanthine dehydrogenase/oxidases (XDH), the flavin adenine dinucleotide (FAD)-dependent polyamine oxidases (PAOs) and the copper amine oxidases (CuAOs), also called diamine oxidases (DAOs), generate ROS [22,23]. CuAOs oxidize Put and Cad at the primary amino groups, producing ammonia, H_2_O_2_, and an aminoaldehyde. The *Arabidopsis thaliana* genome contains 10 CuAO genes [22,24]. CuAOs show tissue-specific expression patterns and localize in different compartments of the cells, such as the apoplast, peroxisomes, or vacuoles [24].

Plant PAOs can be classified into two classes based on their functions in PA catabolism. The first class of PAOs is responsible for the terminal catabolism of PAs. The reaction they catalyse leads to the oxidation of Spd or Spm, which results in the production of H_2_O_2_, 1,3-diaminopropane (DAP), and 4-aminobutanal (in the case of Spd catabolism) or N-(3-aminopropyl)-4- aminobutanal (in case of Spm catabolism). The PAOs in the second class catalyse PAs back-conversion reactions, such as the conversion of Spm to Spd and Spd to Put. These PAOs also generate H_2_O_2_ as a product of their catalytic activity. The PA terminal catabolic pathway is specifically activated extracellularly, whereas the PA back-conversion pathway mainly occurs in the intracellular space in the cytoplasm and mostly in peroxisomes [25]. PAOs may have substrate specificities and tissue-dependent differences in their expression pattern [26,27]. Considering their cellular localization, apoplastic, cytosolic, and peroxisomal PAOs are distinguished [3,22]. Until recently, PAO genes were characterized in both monocots and dicots [4,28]. The best-studied and characterized PAO is an apoplastic PAO (ZmPAO) of maize [29,30]. In *Arabidopsis thaliana*, the PAO isoforms are coded by five PAO genes. AtPAO1 catalyses the oxidation of Spm, [31] while AtPAO3 prefers Spd as substrate [32]. AtPAO2 and AtPAO4 have similar affinity for both Spd and Spm [33]. Interestingly, AtPAO5 is a t-Spm oxidase, as it catalyses the back-conversion of t-Spm to Spd [27]. PAOs achieve different optimal pH values and operation temperatures upon catalysing different reactions [33,34]. Emerging evidence suggests that PAOs and PA catabolic products play a critical signalling role in a variety of cellular and developmental processes [2,4,25]. These roles are likely mediated via the regulation of PA homeostasis, as well as the generation of H_2_O_2_. 

This review would like to highlight a small piece of the complex polyamine signalling network in plants—the role of the polyamine catabolising PAO enzymes in ROS generation and signalling and their interlink with plasma membrane NADPH oxidases in this respect.

## 2. PAOs in Plant Development

There is accumulating evidence suggesting that PAs interfere with various biological processes through the generation of H_2_O_2_ during their catabolism [4,7]. In agreement, the participation of PAOs has been reported in many developmental processes where ROS are involved (Table 1).

### 2.1. PAOs in Cell Differentiation

Overexpression of the maize (*Zea mays*) ZmPAO1 resulted in early xylem differentiation and strongly affected root development in transgenic tobacco plants in correlation with augmented H_2_O_2_ production and increased rate of cell death [44]. This PAO was shown to accumulate in the cell wall of xylem precursors parallel to secondary wall deposition [30]. The AtPAO5 enzyme that specifically accumulates in the vascular system has also been reported to participate in xylem differentiation [45,46]. Although most PAOs generate ROS as a by-product of their activity, AtPAO5 preliminary control PA, especially t-Spm, levels [27,45,46]. Since AtPAO5 acts rather as a dehydrogenase than oxidase, it does not produce excess H_2_O_2_ [45]. In this way, AtPAO5 indirectly controls xylem differentiation via maintaining t-Spm homeostasis required for normal growth [37,45]. AtPAO5, via controlling the t-Spm level, was hypothesized to contribute to the tightly controlled interplay between auxins and cytokinins during the xylem differentiation process [46].

Nevertheless, there is ample evidence suggesting that the production of H_2_O_2_ by PA catabolism contributes to the cross-linking of cell wall polysaccharides during cell wall maturation [30,44]. For example, the apoplastic maize ZmPAO enzyme was shown to provide ROS for peroxidase-mediated wall stiffening during wound healing [47,48]. Moreover, rice OsPAO7 was hypothesized to control lignin synthesis in anther cell walls [49]. The polar growth of pollen tubes correlates with ROS accumulation at their tip region, controlling hyperpolarization-activated Ca^2+^ channels and cell wall stiffening [50]. Exogenous polyamines modulate pollen tube growth dependent on ROS generation [51]. In agreement, Spd treatment was reported to promote the opening of Ca^2+^ channels in pollen tubes [52]. Mutations in the AtPAO3 gene blocked the effect of Spd on Ca^2+^ channels and pollen tube growth, indicating that the effect was dependent on AtPAO3-mediated Spd degradation. Untreated pollen tubes of the AtPAO3 mutant also exhibited retarded growth, supporting the view that AtPAO3-generated ROS contributes to pollen tube growth [52]. The observation that the Arabidopsis polyamine transporter ABCG28 is required for the apical accumulation of ROS in growing pollen tubes and root hairs [53] further strengthens this hypothesis. PAs and their catabolism play a role in the induction of Ca^2+^ and K+ fluxes also in roots during stress adaptation [54], supporting the general significance of the PA-PAO-ROS-Ca^2+^ signalling connection.

PAs are well known to be required for and promote in vitro plant regeneration, although the mechanism is largely uncovered (reviewed by [55]). It was suggested that metabolic degradation products of PAs, such as t-Spm and/or H_2_O_2_, can at least be partly responsible for the observed effects [56]. The expression of AtPAO5, unlike other AtPAO-coding genes, increased in parallel with the conversion of lateral root primordia to shoot meristem during direct in vitro organogenesis [56]. Furthermore, the ectopic expression of AtPAO5 but not AtPAO2 promoted the process. It was hypothesized that AtPAO5 exerted its effect via the modulation of the t-Spm homeostasis rather than H_2_O_2_ production [56] since AtPAO5, unlike the other AtPAO enzymes, is known to have a stronger dehydrogenase than oxidase activity and has a high affinity for t-Spm as substrate [45]. Interestingly, AtPAO5 had been reported to have a negative effect on indirect (via auxin-induced callus formation) [57] and not direct (via cytokinin-induced meristem conversion) [56] shoot regeneration from Arabidopsis roots. This strengthens the view that maintaining t-Spm homeostasis is the primary function of AtPAO5 [27] since t-Spm was shown to suppress auxin signalling [58], which plays a different role in direct and indirect shoot regeneration from Arabidopsis roots [59]. Furthermore, H_2_O_2_ as the metabolic product of PAs was found to be essential for the maintenance and propagation of embryogenic calli and their conversion into somatic embryos in cotton [60], indicating a more general role of PA catabolism in plant regeneration in vitro.

### 2.2. PAOs in Senescence and Programmed Cell Death

The link among PAs, ROS, and leaf senescence has been long established (reviewed in [61]). Augmenting transcription and activity of PA catabolic enzymes has been demonstrated during dark-induced senescence of barley leaves [61,62]. Inhibiting the PAO activity delayed the senescence process in parallel with Spm accumulation and reduced ROS production. In agreement, the Arabidopsis atpao4 mutant exhibited delayed senescence in correlation with high Spm levels but reduced ROS accumulation [38]. Altogether, the observations indicate that PAO-generated H_2_O_2_ is involved in leaf senescence. PA catabolism has been also associated with fruit ripening, a senescence-like developmental process in grapes, tomatoes, and peaches [63,64,65]. Fruit ripening is associated with the increased expression of genes coding for apoplastic PAO enzymes, catalysing the terminal oxidation of PAs. Inhibition of PAO activity reduced ethylene production and flesh softening of peach fruits and the expression of ripening-related genes, while PA contents were dramatically increased. The role of PAO-generated H_2_O_2_ as a ripening-promoting signal molecule was hypothesised as one of the potential mechanisms [22]. PAO-generated H_2_O_2_ was shown to contribute to developmental PCD during xylem differentiation [44,66]. Polyamine oxidases were also found to be key elements in the oxidative burst, leading to programmed cell death in cryptogein-treated tobacco-cultured cells [67]. Moreover, tobacco plants overexpressing the transgene coding for the same maize PAO enzyme had high H_2_O_2_ levels, which in some cases led to programmed cell death (PCD) [68].

### 2.3. PAOs and Abiotic Stress Responses

There is overwhelming evidence that increasing the polyamine content contributes to cell protection under environmental stress conditions. PAs take part in osmoprotection, stabilisation of macromolecular complexes, maintenance of the ion homeostasis, scavenging ROS, and stress and hormone signalling [2,69]. Not only biosynthesis, but PA catabolism has also been shown to have a significant role in various abiotic stress responses [25,70]. These roles can at least partly be attributed to the products of PA catabolism catalysed by DAO and PAO enzymes, such as gamma-aminobutyric acid (GABA) and/or H_2_O_2_ [70]. GABA, which is mainly synthesized in PA-independent pathways but can also be produced from PA-derived 4-aminobutanal, is an important plant metabolite with various protective functions in stress tolerance [71]. Under hypoxic conditions, PA catabolism with CuAO and PAO enzymes contributed to the GABA content by approximately 30% in Vicia faba [72]. Exogenous GABA, however, was shown to inhibit the breakdown of PAs, indicating negative feedback [71]. Besides GABA, the significance of PAO-dependent H_2_O_2_ generation has also been described in drought adaptation, namely during ABA, as well as ethylene-mediated stomatal closure in Vitis vinifera and *Arabidopsis thaliana*, respectively [73,74]. Fine-tuning PA catabolism during stress conditions might be required to control H_2_O_2_ generation. ROS produced by PA decomposing enzymes can serve as important signalling molecules to boost antioxidative defence reactions, but above a certain level, they can augment the stress-associated cellular damage or even lead to PCD [25,75]. For example, tobacco cells were found to secrete Spd into the apoplast where it was oxidized by PAO, thereby generating H_2_O_2_ at a level that promoted PCD [32,68]. In citrus (Citrus sinensis), the apoplastic CsPAO4 was shown to produce H_2_O_2_ and cause oxidative damages under salt stress [41]. In tomato, PA catabolism (both DAO and PAO enzymes) responded stronger to sublethal than lethal doses of the stress hormone salicylic acid in salinity tolerance signalling [76]. Downregulated expression of PAO-coding genes increased the thermotolerance of tobacco likely due to reduced heat-induced H_2_O_2_ generation [77]. PAO activity was shown to contribute to aluminium- or selenium-induced oxidative stress, further strengthening its pro-oxidant role during severe stresses [78,79]. 

However, the significance of PA-catabolism in antioxidant defence signalling contributing to the salt tolerance of PA-overproducing transgenic tobacco plants was also demonstrated by different groups [68,80]. Furthermore, contrasting salt stress tolerance of maize genotypes was found to be correlated with PA catabolism-dependent H_2_O_2_ production during salt stress, but it was rather DAO than PAO activity-dependent [81]. In the leaf blade elongation zone of salinized maize plants, the PAO activity was found to be strongly increased (app. 20-fold) [82]. Together with increased apoplastic PA secretion, the PAO activity resulted in increased apoplastic ROS accumulation, contributing to leaf blade elongation under salt stress. Polyamine oxidase 5 loss-of-function atpao5 mutants of Arabidopsis are salt-stress tolerant; however, their salt tolerance did not show correlation with diminished ROS production but rather with the increased level of t-Spm [37]. However, in the salt-tolerant pao1 pao5 double mutant with no cytoplasmic PAOs, reduced ROS production was observed under NaCl stress [36]. Interestingly, simultaneous mutations in the pao2 and pao4 genes, both coding for peroxisomal PAO, were salt-sensitive, while the pao2 pao3 pao4 triple mutant with no peroxisomal PAO enzymes was not viable [36]. 

The above observations highlight the differential contribution of the various PAOs with different activities, by-products, and intracellular localisations, to stress tolerance.

### 2.4. PAOs in Host–Pathogen Interactions

PAO-generated H_2_O_2_ may also contribute to pathogen defence. It may directly act as an anti-microbial agent in the apoplast or serve as a signalling molecule inducing the activation of defence genes [83,84]. PA levels and the activity of PA metabolic enzymes were found to be induced by various (biotrophic, as well as necrotrophic) pathogens infecting plant tissues [85,86,87]. For example, in response to the biotrophic pathogen *Pseudomonas syringae*, PAO activity was found to be increased in tobacco [88]. The infection also induced Spm secretion that, together with the elevated PAO activity, resulted in strong H_2_O_2_ accumulation in the apoplast. The apoplastic Spm-mediated disease resistance could be compromised by PAO inhibitors [86,88]. Therefore, in biotrophic plant–pathogen interactions, PAO activity-related H_2_O_2_ generation might contribute to the hypersensitive response (HR), as described for tobacco mosaic virus or *Pseudomonas chicorii*-infected tobacco [89,90] and powdery mildew (*Blumeria graminis*)-infected barley [89]. The oomycete *Phytophthora cryptogea* secretes cryptogein, a 10-kD protein that induces HR in tobacco. Inhibiting the expression of the gene coding for apoplastic tobacco PAO prevented PA degradation, cryptogein-induced apoplastic H_2_O_2_ generation, and cell death [67]. The observation that, in these plants, cryptogein-induced kinase signalling was also compromised, highlighted that, besides its cytotoxic effect, PAO-generated H_2_O_2_ also has a signalling role in the HR. The signalling role of Spm degradation-derived H_2_O_2_ was also hypothesized in the transcriptional responses of Arabidopsis to the HR-inducing cucumber mosaic virus [91]. While PA degradation and H_2_O_2_ production might beneficially control the HR response in biotrophic host–pathogen interactions, it might be detrimental in the case of infection by necrotrophic pathogens. In agreement, increased polyamine levels were reported to promote leaf necrosis during fungal infection dependent on PAO activity [86]. 

## 3. The PAO and NADPH-Oxidase Regulation Nexus

NADPH oxidases, the RBOHs, are key enzymes regulating the controlled production of reactive oxygen species (ROS) during plant development and adaptation [92]. PAO and RBOH enzymes are involved in many cellular phenomena, raising the possibility that they are functionally interlinked in the control of ROS homeostasis. Both PAO and RBOH enzymes have a role in pollen tube growth [52,93,94]. The mechanism of acclimation to aluminium stress requires the operation of apoplastic PAO and the RBOH enzymes as well [78]. In *Solanum lycopersicum*, melatonin acts as a signalling molecule to regulate the SlPAO1 and SlRboh3/4 genes during lateral root development, implicating that both PAO and RBOH act downstream of melatonin in this process [95]. Longer uncommon polyamines (LUPAs) activate PAO and induce the expression of RBOH genes [96].

The convergent action of PAOs and RBOHs can be well exemplified by their control of the stomatal aperture. Stress factors, as well as plant hormones, induce stomatal closure via the production of H_2_O_2_ [40,97]. H_2_O_2_ production activates Ca^2+^ channels that are ROS-dependent, thus increasing cytosolic Ca^2+^, triggering the signal transduction cascade and leading to the closure of stomata [98]. This H_2_O_2_ mainly arises from the O_2_•- generated by RBOHs [73,92,99,100]. In maize leaf cells, peroxidase and PAO activities also contributed to ABA-induced H_2_O_2_ generation, although at a lower degree than RBOH [101]. Exogenous PAs Put, Spd, and Spm increase the level of ROS in guard cells and promote stomata closure in Arabidopsis [40]. Application of either diphenyleneiodonium (DPI), an inhibitor of NADPH oxidase, or 2-bromoethylamine (BEA), an inhibitor of copper amine oxidase, or 1,12 diaminododecane (DADD), an inhibitor of polyamine oxidase, could only partially reverse the stomatal closure. DPI, in combination with BEA/DADD, however, completely reverses the closure brought about by PAs. Therefore, the production of ROS during PA-mediated stomatal closure is controlled by both RBOH and amine oxidases. Stomatal closure in response to ethylene was shown to be dependent on AtrbohF-mediated H_2_O_2_ production [73]. Nevertheless, the use of PAO inhibitors on Arabidopsis epidermal peels hindered ethylene’s ability to stimulate H_2_O_2_ production and stomatal closure [102]. In agreement, ethylene induces AtPAO2 and AtPAO4 gene transcription and PAO activity. Furthermore, the over-expression of AtPAO2 and AtPAO4 in Arabidopsis plants led to increased production of H_2_O_2_ and higher sensitivity of stomatal movement to ethylene [102]. Other factors which induce stomatal closure, such as dehydration and high salinity, enhanced the expression of AtPAO2 and AtPAO4 to different degrees, indicating a general role of PAO-generated H_2_O_2_ production as a stress-induced stomatal response [102]. The above observations support the view that, although a majority of H_2_O_2_ is produced by RBOH enzymes in response to stomata-closing conditions, the contribution of PAOs cannot be neglected. A similar conclusion was drawn when investigating the oxidative burst associated with hyperhydricity in vitro in cultures of garlic where RBOH activity was more prominent than that of PAO [103].

RBOH are also key players in the wound and jasmonic acid responses of plants [104,105,106]. The inhibition of MeJA-induced ROS production by treatment with DPI was observed in tomato, rice, and pea plants [107,108,109]. Pre-treatment with DPI or a lack of AtRbohD or AtRbohF almost entirely prevented the accumulation of H_2_O_2_ in Arabidopsis [110]. However, in maize, apoplastic polyamine oxidase (ZmPAO) was reported as the main producer of ROS in response to MeJA and wounding [47]. The researchers used N-prenylagmatine (G3), a specific and selective ZmPAO inhibitor, to study its effects on wound-induced cell wall lignification and suberinization in vivo. In addition, they looked at transgenic tobacco plants that constitutively express high levels of ZmPAO in their cell walls. G3 significantly inhibited lignin and suberin deposition in the wound periderm of maize mesocotyls. Furthermore, ZmPAO overexpression accelerated the same process in wounded tobacco stems, especially if the plants were treated with the ZmPAO substrate spermidine. Spd enhanced lignosuberized deposition in the cell walls of wild-type tobacco as well, suggesting that an endogenous amine oxidase might be involved in wound-healing processes not only in maize but in tobacco plants as well. Further, experimental evidence indicates that CuAOs also participate in the wound response [47]. Therefore, the degree of contribution of various enzymes to wound-induced H_2_O_2_ generation might be species-specific [47].

The above examples highlighted the correlated action of PAO and RBOH enzymes in several plant responses. However, there are many studies supporting the hypothesis that PAO and RBOH activities are not simply correlated but are interconnected and can impact each other. 

There is ample evidence that suggests that exogenous PAs alter the transcription of RBOH genes and/or the activity of the RBOH enzymes (Table 2). In tobacco leaf protoplasts, exogenous PAs were shown to reduce the accumulation of superoxide anions (O_2_•-) likely generated by microsomal NADPH oxidase during tissue maceration [111]. Andronis et al. [39] discovered that exogenous PAs, especially Spd, increased oxygen consumption through an NADPH-oxidase-dependent mechanism. The NADPH-oxidase blocker DPI attenuated the increase. The loss of function of the AtPAO3 gene resulted in the increased production of O_2_•- through NADPH oxidase, which in turn activated the mitochondrial alternative oxidase pathway (AOX). Overexpression of AtPAO3 led to an increased but balanced production of both H_2_O_2_ and O_2_•-. These observations indicate that the ratio of O_2_•- to H_2_O_2_ controls the respiratory chain in mitochondria, and PAO-dependent production of O_2_•- by NADPH-oxidase alters this ratio in favour of the AOX pathway of the electron transfer chain. 

Seo et al. [80] found that the expression of NtRbohD and NtRbohF genes was reduced under NaCl stress conditions in S-Adenosyl-L-Methionine Decarboxylase (SAMDC) overexpressing *Nicotiana tabacum* plants with upregulated PA content. Thus, they determined that polyamines interfere with the production of ROS through RBOH enzymes. Treatment of cucumber (*Cucumis sativus* L.) plants with Spd decreased the activity of NADPH oxidases and NADPH-dependent O_2_•- generation in microsomes, alleviating H_2_O_2_ generation and injury under chilling stress [112]. Inhibiting PA biosynthesis enhanced microsomal NADPH oxidase activity and chilling injury in stressed plants. In agreement, the direct inhibitory effect of Spd and Spm on the activity of a *Lotus glaber* NADPH oxidase in vitro and on O_2_•- generation in vivo was also demonstrated [113]. There are also examples where polyamines or polyamine degradation enhanced RBOH-dependent ROS generation. The salinity–alkalinity stress tolerance of tomato seedlings could be increased by exogenos Spd via RBOH1-dependent H_2_O_2_ generation [117]. Exogenous polyamines increased the expression of RBOH-coding genes and the NADPH oxidase activity in apricot fruits, limiting the oxidative damage caused by Alternaria alternata. Gémes et al. [42] hypothesized that apoplastic PAO activity controls that of RBOH to amplify ROS generation in a positive feedback loop. These observations implicate that the activation of RBOH gene transcription, enzyme activity, and thus, RBOH-mediated ROS generation is controlled by polyamine metabolism. 

There are, however, observations supporting the hypothesis that RBOH activity plays a role in regulating the metabolism of polyamines. Demiralay et al. [118] studied polyamine metabolism in detail after the application of H_2_O_2_ or an RBOH inhibitor to drought-stressed maize plants. It was found that inhibition of the RBOH enzyme by DPI enhanced polyamine degradation while exogenous H_2_O_2_ promoted their synthesis, and RBOH played a key role in that regulation [118]. The observation that exogenous H_2_O_2_ increased while DPI decreased the expression of the arginine decarboxylase (ADC) and agmatine aminohydrolase (AIH) genes, which encode enzymes involved in Put synthesis, suggests that H_2_O_2_ produced by RBOH may contribute to the regulation of polyamine biosynthesis [118]. The expression level of DAO- and PAO-coding genes was higher in DPI-treated and lower in H_2_O_2_-treated than in control plants, supporting the idea that RBOH also controls polyamine degradation. 

The RBOH–PAO crosstalk was also demonstrated in the *Arabidopsis thaliana*—*Pseudomonas syringae* pathosystem [35]. Pseudomonas infection upregulates the transcription of AtPAO1 and AtPAO2 genes, and the double mutant atpao1-1 × atpao2-1 has increased susceptibility to the pathogen. The polyamine oxidases mutant showed not only disturbed H_2_O_2_ but also O_2_•- generation, which could be associated, among others, with the increased activity of RBOH enzymes. The lower expression levels of AtRbohD, AtRbohF genes in the mutant background were also reported. It was, therefore, hypothesized that peroxisomal Spm oxidation by PAOs negatively regulates RBOH activity in Arabidopsis by an unknown mechanism that could involve H_2_O_2_ signalling [35].

Yoda et al. studied ROS generation during cryptogein-induced cell death in tobacco cell culture [67]. Co-treatment of the cells with cryptogein and a-difluoromethylornithine (DFMO), an irreversible inhibitor of polyamine synthesis via the ornithin decarboxylase enzyme, effectively suppressed H_2_O_2_ production and prevented cell death. However, DFMO hardly had any influence on cryptogein-induced O_2_•- production during the first 4 h of the elicitation of cells. The results suggested that at least two systems are involved in cryptogein-induced programmed cell death featuring RBOH in the early and polyamine degradation at the late stage.

The findings presented by Gémes et al. [42] suggest that a feed-forward loop involving apoplastic PAO and RBOH controls ROS accumulation in response to salt stress in tobacco. RBOH was found to be required for ROS production induced by NaCl exposure in the early stages, while that of PAO was dispensable at this stage. The subsequent activation of apoplastic PAO was hypothesized to amplify ROS accumulation, thereby enhancing RBOH activity. At deleterious salt concentrations, this apoplastic PAO-fed ROS amplification loop causes the accumulation of ROS that surpasses a toxicity threshold, resulting in PCD.

## 4. Coordination of PAO and NADPH-Oxidase Activities

**Figure 1:**
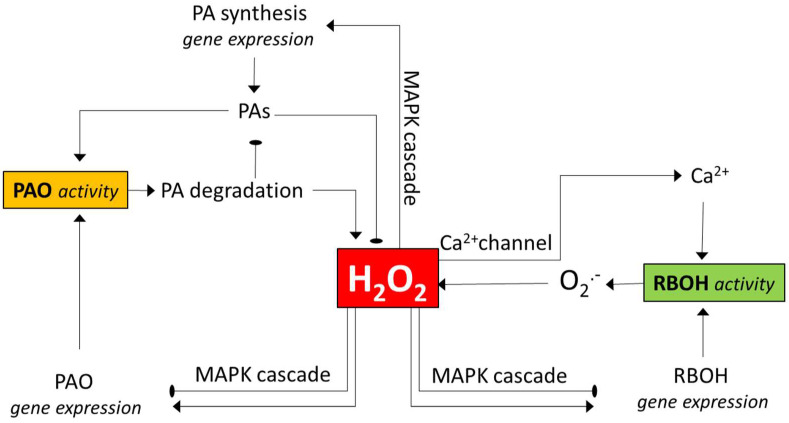
An outline of the pathways of how polyamine oxidase (PAO) and NADPH oxidase (RBOH) enzymes may influence each other’s activity. Both enzymes contribute to H_2_O_2_ generation. H_2_O_2_ may activate MAPK signalling, thereby leading to altered expression of the genes for any or both enzymes. The changes in gene expressions can either be positive or negative, depending on the context (see the text for examples). H_2_O_2_ can also activate genes involved in polyamine biosynthesis, and polyamines can serve as PAO substrates generating H_2_O_2_ or as H_2_O_2_ scavengers. H_2_O_2_ can also open Ca^2+^ channels, and the increased intracellular Ca^2+^ level augments the activity of the RBOH. Note that the various interactions are context-dependent, and several other ways of more indirect interactions may exist between the enzymes (e.g., via the activation of ROS-detoxifying mechanisms). PAO—polyamine oxidase; PAs—polyamines; MAPK—mitogen-activated protein kinase; RBOH—respiratory burst oxidase homolog (NADPH oxidase). The pointed arrows indicate the activation the round-pointed ones the inactivation.

It is unclear how the activities of PAO and RBOH might be interconnected since the experimental data derived from various species and experimental systems are not fully consistent. However, the available data allows us to hypothesize some ways for their potential interaction (Figure 1).

Yoda et al. [67] hypothesized, based on their observations with cryptogein-elicited tobacco cell cultures, that H_2_O_2_ produced during the early phase of elicitation by RBOH activates a MAPK cascade, including SIPK that is required to provoke the second phase of H_2_O_2_ generation by PAO [67]. Similar timing of RBOH and PAO activities has been observed in other experimental systems, suggesting that it is a rather common scenario (see earlier). However, the effect of RBOH-generated H_2_O_2_ on PAO gene expression is controversial, since H_2_O_2_ application was shown to decrease while DPI increased PAO gene expression in maize seedlings [118]. RBOH activity can enhance PA synthesis [118], and the elevated PA level may contribute to increased PAO activity [40,118]. The other way around, the effect of PAO activity on RBOH-mediated ROS generation, is more complex. A feed-forward loop linking RBOH and PAO activities was hypothesized to establish during lethal salt stress, leading to uncontrolled H_2_O_2_ generation and PCD in tobacco [42]. In this scenario, PAO-generated H_2_O_2_ could open Ca^2+^ channels and thereby increase the activity of Ca^2+^-regulated RBOH enzymes. PAO-mediated regulation of Ca^2+^ channels was demonstrated in other systems, such as in pollen tubes, where PAO and RBOH enzymes are both required for tip growth [52,93,119]. There are reports, however, where mutations in Arabidopsis AtPAO1- and AtPAO2-coding genes resulted in enhanced O_2_•- generation and RBOH activity [35,39], indicating the negative effect of PAO action on that of RBOH. This can partly be explained by the AtPAO-dependent altered expression of some of the AtRboh genes. Interestingly, however, while the expression of the investigated AtRbohD and AtRbohF genes was increased in the atpao2-1 mutant, it was decreased in the double atpao1-1 atpao2-1 mutant. Gémes et al. studied the effect of an apoplastic PAO enzyme in tobacco [42] and found that the AtPAO1 and AtPAO2 [35] and AtPAO3 [39] enzymes are cytosolic and peroxisomal, respectively. Thus, PAO enzymes might have an intracellular localisation-dependent effect on RBOH gene expression and/or activity. Again, further studies dissecting the specific roles of PAO isoenzymes are required to clarify the picture.

The existence and the exact nature of the hypothesized RBOH–PAO regulatory loops still need further verification, as it can lead to a better understanding of how plant cells can fine-tune H_2_O_2_ generation to ensure their proper functioning, survival, or programmed death depending on the developmental/environmental context.

## 5. Conclusions and Future Perspectives

Although PAO enzymes can have different expression, biochemical activity, and intracellular localisation and may exhibit species–specific differences in these parameters, they all contribute to the generation of H_2_O_2_. Even though H_2_O_2_ is only a by-product of the polyamine degradation activity of PAOs, accumulating evidence shows that PAOs control various cellular processes via H_2_O_2_-mediated pathways. This review attempted to summarize the current knowledge about these pathways. It is to be emphasized, however, that PAO action is more diverse. Besides H_2_O_2_ generation, PAO activity alters polyamine levels and ratios and can contribute to the generation of regulatory metabolites / signalling molecules such as t-Spm, GABA, and NO. Since the interrelation of these PAO activities and their contribution to cellular functions is rather complex, we restricted our focus to H_2_O_2_ generation-dependent mechanisms. PAO-generated H_2_O_2_ is a two-edged sword; it is required for normal development and can enhance stress adaptation, but it can also be harmful and lead to cell death if it exceeds a threshold level. Stress-induced or developmentally regulated augmented biosynthesis or exogenous application of higher polyamines (Spm or Spd) often induces PAO activity. PAs thus can exert a concentration-dependent positive or negative effect on cellular functions via their degradation during which H_2_O_2_ is produced. H_2_O_2_ may serve as a signalling molecule to alter Ca^2+^ homeostasis, MAPK signalling, and gene expression-modifying cellular processes such as growth, division, differentiation, and adaptation. The contribution of PAO-generated H_2_O_2_ to these processes is hard to define since the actual level of H_2_O_2_ is controlled by many other enzymes, including producers (RBOH, superoxide dismutase, CuAO) and removers (catalase, peroxidases), as well as non-enzymatic pro- and anti-oxidants. Future research should unravel the details of how PAO activity is integrated into the cellular machinery that controls ROS homeostasis. The isoenzyme-specific spatial (apoplast, cytoplasm, peroxisome) and temporal (sequence of events) contexts of this integration deserve special attention.

## Figures and Tables

**Table 1:** Mutations in plant polyamine oxidase-coding genes affecting reactive oxygen species homeostasis and polyamine levels under various experimental conditions. The cell compartements where the mutations prevented the accumulation of the given polyamine oxidases are indicated. At—*Arabidopsis thaliana*; Cs—*Cucumis sativus*; Zm—*Zea mays*; Spd—spermidine; Spm—spermine; t-Spm—thermospermine; Put—putrescine; *pao/PAO*—polyamine oxidase; RBOH—Respiratory Burst Oxidase Homolog; PRX—peroxidase; CAT—catalase; APX—ascorbate peroxidase; SOD—superoxide dismutase; GABA—gamma aminobutyric acid; NA—not applicable.

Organism	Mutant Line and ID Number	Condition and Cell Compartment	Alteration of ROS Levels	Change in PA Levels	Change in ROS Related Enzyme Activities or Gene Expression	Ref.
*Arabidopsis* *thaliana*	*atpao1-1* (SALK_013026.56.00)	Pseudomonas infection cytoplasm	increase in H_2_O_2_ and O_2_•-	increased Put decreased Spm	increased RBOH, and CAT activity increased *AtPRX33* and *AtPRX34* expression	[35]
	*atpao2-1* (SALK049456.42.05.)			increasedPut		
	*atpao1-1* × *Atpao2-1* (genetic crosses)		H_2_O_2_ and O_2_•-similar to control	increased Put	increased RBOH, and CAT activity	
	*atpao1-2* (SAIL_822_A11)	salt and drought stress cytoplasm	not changed	NA	NA	[36]
	*atpao5-2* (SALK_053110)			increased t-Spm		
	*atpao1* × *Atpao5* (genetic crosses)		decrease in H_2_O_2_ and O_2_•-		increased CAT and PRX activity	
	*atpao5-2* (N553110)	salt stress cytoplasm	no change in ROS	increased t-Spm	NA	[37]
	*atpao5-3* (N509671)					
	*atpao4-1* (SALK_109229)	dark induced senescence peroxisome	decrease in H_2_O_2_; increase in NO	increased Spm decreased Spd	enhancement of the antioxidative machinery, GABA accumulates	[38]
	*atpao4-2* (SALK_133599)	senescence peroxisome				
	*atpao3-1* (Salk 121288)	seedling development peroxisome	increase in O_2_•- and decrease in H_2_O_2_	increased Spd	increased RBOH APX activity increased expression APX SOD	[39]
	*S-AtPAO3* (overexpressing AtPAO3 cDNA)		increase in H_2_O_2_ and O_2_•-	NA	reduced expression of APXincreased expression SOD	
	*atpao5-1* (SAIL_664_A11)	plant growth cytoplasm	not changed	increased t-Spm	NA	[27]
	*atpao5-2* (SALK_053110)					
	*atcuao1-1* (SALK_019030CC)	stomatal closure peroxisome	small decrease in H_2_O_2_ and NO	NA	NA	[40]
	*atpao4* (SALK_133599C)				enhancement of the antioxidative machinery	
*Nicotiana tabacum*	*CsPAO4* overexpression	salt stress peroxisome	increase in H_2_O_2_ and decrease in O_2_•-	decreased Spm and Spd	NA	[41]
	*ZmPAO* overexpression	salt stress apoplast	increase in H_2_O_2_ and O_2_•-		increased RBOH D, F and APX, SOD expression	[42,43]

**Table 2:** The effect of exogenous polyamine application on NADPH oxidase level/activity. Spd -spermidine; Spm—spermine; Put—putrescine; *Rboh*—Respiratory Burst Oxidase Homolog gene.

Organism	PA Treatment	Condition or Process	Change in NADPH Oxidase Activity or Expression	References
*Cucumis* *sativus*	1 mM Spd	chilling stress	inhibited activity	[112]
*Triticum* *aestivum*	2 mM Put	roots under aluminium stress	inhibited activity	[78]
*Nicotiana* *tabacum*	2.5/5 mM Put/Spd/Spm	protoplast regeneration	inhibited activity	[111]
*Nicotiana* *tabacum*	0.2 mM Put/Spd/Spm	salt stress	increased expression of *NtRbohD* decreased expression of *NtRbohF*	[80]
*Lotus* *glaber*	1 mM Spd/Spm	herbicide, methyl viologen stress	inhibited activity	[113]
*Arabidopsis* *thaliana*	0.2/0.5 mM Spd,	seedlings	increased activity	[39]
*Prunus* *armeniaca*	1.5 mM Spm, 1.5 mM Spd or 10 mM Put	fruit’s resistance to Alternaria alternata	increased activity and gene expression	[114]
*Arabidopsis* *thaliana*	100 µM Put	response to pathogen	increased gene expression	[115]
*Cucumis* *sativus*	1 mM Spd	salt stress	increased gene expression	[116]
